# Prognostic risk assessment model and drug sensitivity analysis of colon adenocarcinoma (COAD) based on immune-related lncRNA pairs

**DOI:** 10.1186/s12859-022-04969-4

**Published:** 2022-10-18

**Authors:** Zezhou Hao, Pengchen Liang, Changyu He, Shuang Sha, Ziyuan Yang, Yixin Liu, Junfeng Shi, Zhenggang Zhu, Qing Chang

**Affiliations:** 1 School of Health Science and Engineering, University of Shanghai for Science and Technology, Shanghai, 200093 China; 2 School of Microelectronics, Shanghai University, Shanghai, 201800 China; 3 Shanghai Key Laboratory of Gastric Neoplasms, Department of Surgery, Shanghai Institute of Digestive Surgery, Ruijin Hospital, Shanghai Jiao Tong University School of Medicine, Shanghai, 200020 China; 4 Clinical Research Center, Jiading District Central Hospital Affiliated Shanghai University of Medicine & Health Sciences, Shanghai, 201800 China; 5 Department of Prosthodontics, Shanghai Engineering Research Center of Advanced Dental Technology and Materials, Shanghai Key Laboratory of Stomatology & Shanghai Research Institute of Stomatology, National Clinical Research Center for Oral Diseases, Shanghai Ninth People’s Hospital, College of Stomatology, Shanghai Jiao Tong University School of Medicine, 639 Zhizaoju Road, Shanghai, 200011 China

**Keywords:** Colon adenocarcinoma, Immune-related lncRNA pairs, Prognostic risk assessment model, Drug sensitivity analysis

## Abstract

**Purpose:**

The aim of this study was to identify and screen long non-coding RNA (lncRNA) associated with immune genes in colon cancer, construct immune-related lncRNA pairs, establish a prognostic risk assessment model for colon adenocarcinoma (COAD), and explore prognostic factors and drug sensitivity.

**Method:**

Our method was based on data from The Cancer Genome Atlas (TCGA). To begin, we obtained all pertinent demographic and clinical information on 385 patients with COAD. All lncRNAs significantly related to immune genes and with differential expression were identified to construct immune lncRNA pairs. Subsequently, least absolute shrinkage and selection operator and Cox models were used to screen out prognostic-related immune lncRNAs for the establishment of a prognostic risk scoring formula. Finally, We analysed the functional differences between subgroups and screened the drugs, and establish an individual prediction nomogram model.

**Results:**

Our final analysis confirmed eight lncRNA pairs to construct prognostic risk assessment model. Results showed that the high-risk and low-risk groups had significant differences (training (n = 249): *p* < 0.001, validation (n = 114): *p* = 0.022). The prognostic model was certified as an independent prognosis model. Compared with the common clinicopathological indicators, the prognostic model had better predictive efficiency (area under the curve (AUC) = 0.805). Finally, We have analysed highly differentiated cellular pathways such as mucosal immune response, identified 9 differential immune cells, 10 sensitive drugs, and establish an individual prediction nomogram model (C-index = 0.820).

**Conclusion:**

Our study verified that the eight lncRNA pairs mentioned can be used as biomarkers to predict the prognosis of COAD patients. Identified cells, drugs may have an positive effect on colon cancer prognosis.

**Supplementary Information:**

The online version contains supplementary material available at 10.1186/s12859-022-04969-4.

## Introduction

Colorectal adenocarcinoma (COAD) is a common malignancy of the digestive tract in the colon. It is the second leading cause of cancer mortality [1]. This malignant tumor is highly aggressive and metastatic [2]. The five-year survival rate of patients with advanced colon cancer is poor [3], and the tumor often relapses after surgical resection. An estimated 693,900 deaths and 1.4 million newly diagnosed cases of colon cancer are reported each year [4]. At present, the traditional treatments include surgery, chemotherapy, and radiotherapy, but they offer no obvious improvement in survival rates for colon cancer patients. In recent years, the emergence of molecular targeted drugs like the EGFR (Epidermal Growth Factor Receptor) monoclonal antibody has shown obvious curative effects in patients with terminal colorectal cancer. The median survival time with this treatment has reached 2 years, but the occurrence of anti-EGRF monoclonal antibody resistance caused by *KRAS* (Kirsten Rat Sarcoma) and other mutations can cause a significant reduction in the therapeutic effect of this targeted drug. Therefore, there is an urgent need to understand the molecular mechanisms of colon cancer and to find new therapeutic targets and therapies. With the discovery of the role of lncRNAs (long non-coding RNA) as biomarkers, a series of researchers began to focus on lncRNA.

LncRNA is a molecule lacking protein-coding potential [5]. lncRNA with over 200 nucleotides can be modified and interact with a variety of genes and proteins [6]. Because they may play a role in the underlying pathogenesis, many lncRNAs have been identified as oncogenes or tumor suppressor genes associated with carcinogenesis [7] for cancers of the digestive tract, the urinary tract, lung, breast, and hematopoiesis. LncRNAs show critical processes such as antigen exposure, recognition, and immune osmosis [8]. Therefore, the potential of immune-related lncRNAs in predicting tumor progression and prognosis has attracted increasing attention, and several studies have shown that lncRNAs can be potential biomarkers of COAD prognosis [9]. In addition, the Proportional Hazards Model is often used as the prognostic model of tumors. The proportional Hazards model takes survival outcome and survival time as the dependent variables and can simultaneously analyze the influence of many factors on survival time. We used this method to establish a prognostic model of colon cancer.

In the treatment of cancer, drug therapy is a very important link. Therefore, we used the prognostic models to predict effective prognostic drugs for colon cancer. Paul Geeleher et al. [10] used the expression matrix of the CGP (Cancer Genome Project) database [11], successfully developed a model to predict clinical drug response in patients using baseline gene expression levels and in vitro drug sensitivity of cell lines. The CGP database consists of baseline gene expression microarray data, collected before drug treatment, and sensitivity to 138 drugs in a panel of almost 700 cell lines. This study used the database to study the sensitivity of colon cancer drugs.

Considering the individual differences of patients, we will use the Nomogram model. This model can analyze the values of multiple variables and predict a certain clinical outcome or the probability of certain events. Dong et al. [12] used the Nomogram model to individually predict the 2-week and 3-week survival of COVID-19 patient. Therefore, in order to better facilitate clinical decision-making, this study established an individual prognostic model.

The purpose of this study is to establish a prognostic model for colon cancer and to explore and identify drugs that have been used or may be used for the treatment of colon cancer based on this model.

## Results

### Establishment of a prognostic risk score model based on immune-related lncRNA pairs in patients with colon cancer

**Fig. 1:**
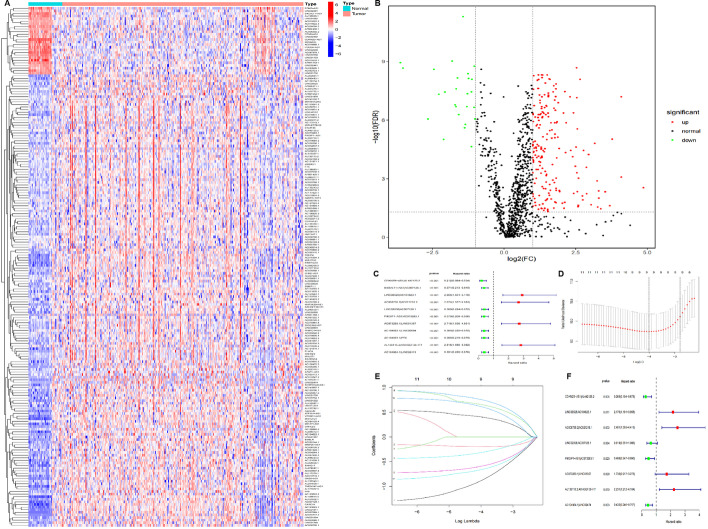
Establishment of a prognostic risk model for immune-associated lncRNA pairs. **A** Heatmap. **B** Volcano plot. Red areas represent upregulated lncRNAs, and green areas represent downregulated lncRNAs. **C** Eleven prognostic related lncRNA pairs screened out by univariate Cox analysis. **D** Tuning parameter (λ) selection in the LASSO model. The Partial Likelihood Deviance was plotted versus log (λ). Dotted vertical lines were drawn at the optimal values by using the minimum criteria and one standard error of the minimum criteria (the 1-SE criteria). A λ value of 0.062, with log (λ), − 4.006. **E** Lasso coefficient profiles of 11 lncRNA pairs. A coefficient profile is generated from the log (λ) sequence. The vertical line is plotted with the determined penalty value, resulting in nine non-zero coefficients. **F** Eight lncRNA pairs were obtained through multivariate Cox analysis for the establishment of a prognostic risk assessment model

Transcriptome data and clinical data of patients with COAD were collected from TCGA (The Cancer Genome Atlas) database. Next, transcriptome data were processed to convert Ensembl ID into gene name. After this conversion, transcriptome data were divided into lncRNA and mRNA. We used immune gene files in the IMMPORT database to combine with gene expression levels in colon cancer patients, the expression level of 1711 immune genes in all colon cancer patients was further extracted. Based on the correlation analysis of lncRNA and immune gene expression, 1229 lncRNAs related to immune genes were identified (Additional file 1). A total of 226 lncRNAs with differences were calculated (Fig. 1A, B). In order to make the model more universal, we did not use a single lncRNA, but paired lncRNAs to produce results by the high and low of expression level. Finally, a total of 16232 lncRNA pairs were calculated. In the training set, through univariate Cox analysis of 16232 lncRNA pairs, 11 lncRNA pairs that were related to pre-post were screened out, including CDKN2B-AS1|AL442125.2, B4GALT1-AS1|AC007128.1, LINC00525|AC104823.1, AC008735.2|AC021218.1, LINC02038|AC007128.1, PIK3IP1-AS1|AC073283.1, AC073283.1|LINC01357, AC104823.1|LINC00894, AC104823.1|FTX, AL136115.2|ARHGEF38-IT1, and AL136115.2|ARHGEF38-IT1 (Fig. 1C).

The above lncRNAs related to prognosis were further analyzed by LASSO (Least Absolute Shrinkage and Selection Operator), and nine lncRNA pairs were screened out (Fig. 1D, E). A further eight lncRNA pairs were screened out by multivariate Cox step-wise regression analysis, including CDKN2B-AS1|AL442125.2, LINC00525|AC104823.1, AC008735.2|AC021218.1, LINC02038|AC007128.1, PIK3IP1-AS1|AC073283.1, AC073283.1|LINC01357, AL136115.2|ARHGEF38-IT1, and AC104964.1|LINC02474 (Fig. 1F). The coefficients of each lncRNA pair in the prognostic risk calculation formula were obtained by the Cox regression model. Therefore, we established a prognostic risk scoring model. The formula is the prognosis risk score (Table 1) = exp (CDKN2B-AS1|AL442125.2 × (− 1.328) + LINC00525|AC104823.1 × 0.775 + AC008735.2|AC021218.1 × 0.909 + LINC02038|AC007128.1 × (− 0.482) + PIK3IP1-AS1|AC073283.1 × (− 0.758) + AC073283.1|LINC01357 × 0.550 + AL136115.2|ARHGEF38-IT1 × 0.802 + AC104964.1|LINC02474 × (− 0.840)).

**Table 1:** The results of multivariate Cox regression coefficients

LncRNA pairs	Coefficients	HR	HR 95% low	HR 95% high	*p* value
CDKN2B-AS1|AL442125.2	− 1.328	0.265	0.104	0.675	0.005
LINC00525|AC104823.1	0.775	2.170	1.191	3.955	0.011
AC008735.2|AC021218.1	0.909	2.481	1.395	4.415	0.002
LINC02038|AC007128.1	− 0.482	0.618	0.351	1.086	0.094
PIK3IP1-AS1|AC073283.1	− 0.758	0.468	0.247	0.890	0.020
AC073283.1|LINC01357	0.550	1.733	0.917	3.275	0.090
AL136115.2|ARHGEF38-IT1	0.802	2.231	1.212	4.104	0.010
AC104964.1|LINC02474	− 0.840	0.432	0.246	0.757	0.003

*HR* Hazard ratio

### Clinicopathologic characteristics of the high-risk group and the low-risk group determined by the model

**Fig. 2:**
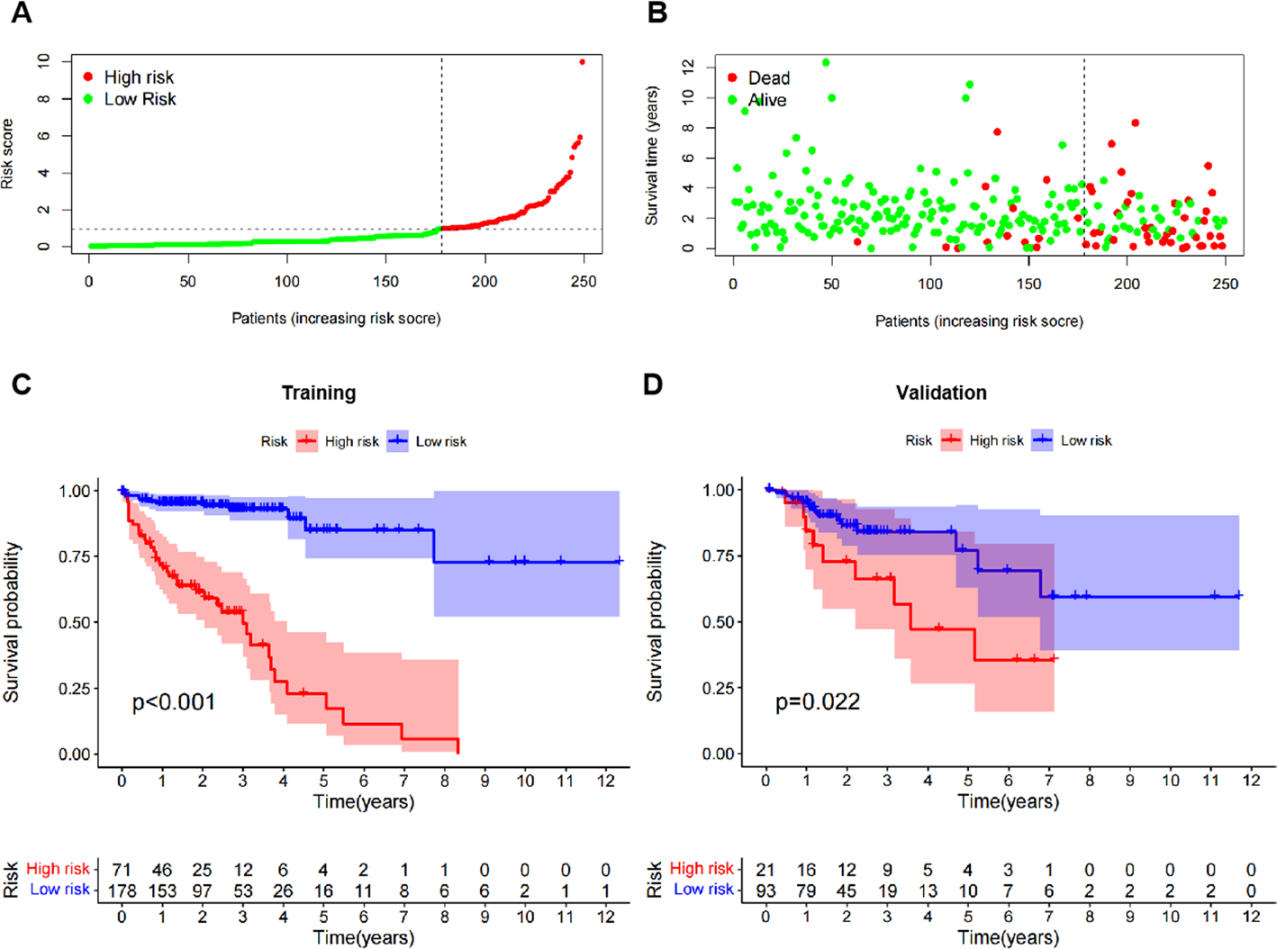
Differences in the high-risk group and the low-risk group. **A** Prognostic risk score distribution diagram of colon cancer patients. **B** Distribution of patients' survival status and survival time. **C** The training set. Kaplan–Meier survival curve of colon cancer patients from the low-risk group and the high-risk group. The high-risk group showed a poorer prognosis. **D** The validation set

To determine the feasibility of the model, the optimal risk score was calculated using the Youden index and was used to separate high-risk groups from low-risk groups (Youden index = 0.979). The training data set was processed, and the samples with no survival time or survival status were removed (n = 16). The training colon cancer samples were divided into a high-risk group (n = 71) and a low risk group (n = 178) (Fig. 2A). Mortality increased with higher risk scores (Fig. 2B). Survival analysis showed that there were statistically significant differences between the groups (*p* < 0.001). The median survival time of the high-risk group (3 years) was significantly shorter than that of the low-risk group (without median survival time) (Fig. 2C). To further validate the model, the validation set of patients can be divided into a high-risk group (n = 21) and a low-risk group (n = 93) using the prognostic risk score formula established by the model. The prognostic analysis found that the high-risk group (median survival 3.58 years) and the low-risk group (without median survival time) also showed a difference in statistical learning significance (*p* = 0.022) (Fig. 2D). These results suggest that the prognostic risk assessment model can effectively predict the prognosis of patients with colon cancer.

### The relationship between model and clinical indicators and the independence of model

**Fig. 3:**
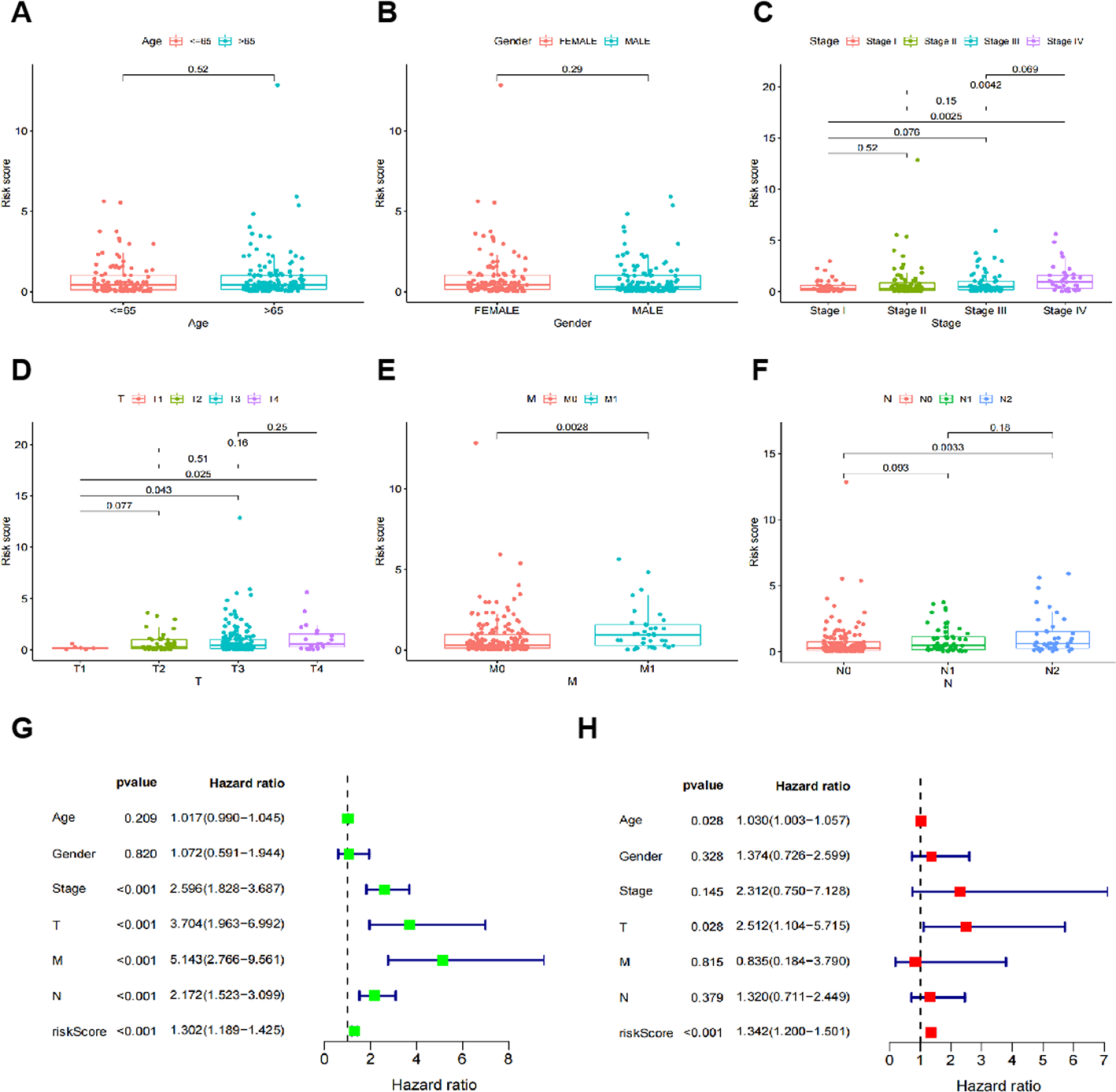
The relationships and independence between the risk score and different clinicopathological features. Relationships between the risk score and age (**A**), sex (**B**), stage (**C**), T-stage (**D**), M-stage (**E**), and N-stage (**F**). Univariate and multivariate Cox analyses to identify prognostic factors in patients with colon cancer. **G** Univariate Cox analysis. **H** Multivariate Cox analysis

To investigate the relevance of the lncRNA pairs and clinicopathological features of risk score, we analyzed the correlation between the risk score and clinical and demographic characteristics, including age (Fig. 3A), gender, stage, T-stage, N-stage, and M-stage. Under the immune-related risk score, women have slightly higher scores than men (Fig. 3B), and the scores of patients with advanced stage (Fig. 3C), advanced T-stage (Fig. 3D), advanced M-stage (Fig. 3E), and advanced N-stage (Fig. 3F) were all significantly increased.

To further confirm the validity of the risk model, a univariate Cox analysis was performed on patients' prognostic risk scores and other related clinical characteristics (age, sex, stage, T stage, M stage, and N stage). Results included: diagnosed stage (HR (Hazard Ratio) = 2.596, 95% CI (confidence interval) 1.828–3.687, *p* < 0.001), T-stage (HR 3.704, 95% CI 1.963–6.992, *p* < 0.001), M-stage (HR 5.143, 95% CI 2.766–9.561, *p* < 0.001), N-stage (HR 2.172, 95% CI 1.523–3.099, *p* < 0.001), and prognostic risk score (HR 1.302, 95% CI 1.189–1.425, *p* < 0.001) all as associated risk factors for prognosis (Fig. 3G). Subsequent multivariate Cox analysis showed that only patients' prognostic risk scores were an independent prognostic risk factor (HR 1.342, 95% CI 1.200–1.501, *p* < 0.001) (Fig. 3H). These results suggest that the risk assessment model can be used as an independent prognostic model for colon cancer compared with conventional clinical features.

### Superiority of the model

**Fig. 4:**
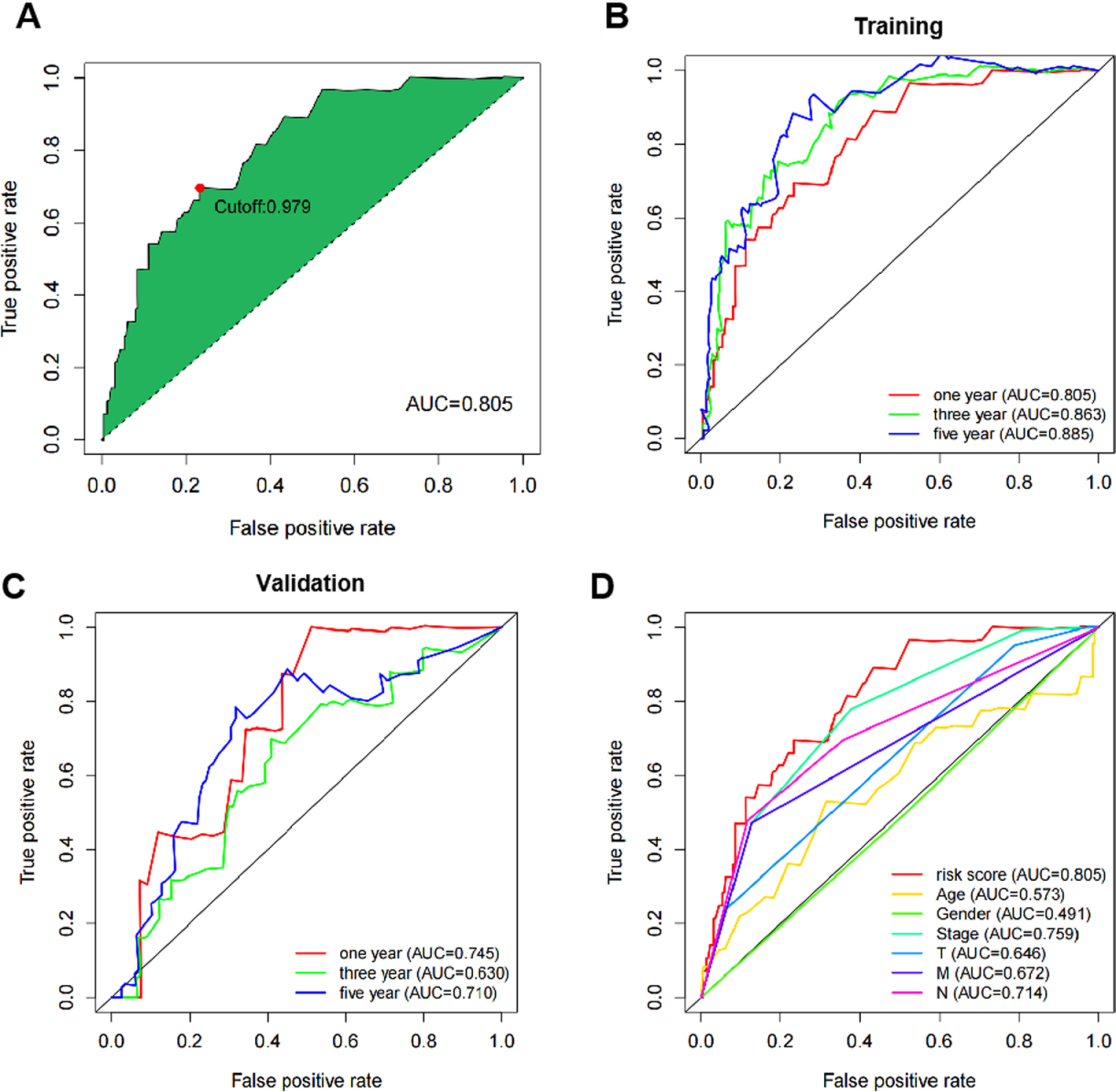
Prognostic risk model of colon cancer patients and prognostic prediction ROC curve of clinically relevant pathological information. **A** Training set prediction results and cut-off. **B** Prediction results of the training set for 1, 3, and 5 years. **C** Verification set prediction results. **D** Prognostic risk model of colon cancer patients and prognostic prediction results of clinically relevant pathological information

In order to show the prognostic ability of risk score, we used ROC (Receiver Operating Characteristic) curve to determine. We use the ROC curve to plot the Youden index (Fig. 4A), and then ROC curves for three consecutive years were drawn with us. The training set showed that the prognostic ability of the model showed a significant upward trend with the increase of time. The AUC (Area Under Curve) for 1, 3, and 5 years was 0.805, 0.863, and 0.885, respectively (Fig. 4B). The validation set AUC was 0.745, 0.630, 0.710 (Fig. 4C).To further verify the predictive ability of the prognostic risk model based on 8 immune-related lncRNA pairs, the ROC curve was drawn and compared with common clinically related pathological information. The AUC corresponding to each indicator was calculated separately. Through comparison, it was found that the predictive power of the prognostic risk model was higher than that of the common clinical characteristics (age, sex, stage, T stage, N stage, and M stage) (Fig. 4D). The above results suggest that the prognostic risk model can effectively predict the prognosis of patients with colon cancer, and the predictive power gradually improves with the extension of time.

### Analysis of functional differences between the high-risk group and the low-risk group determined by the model

**Fig. 5:**
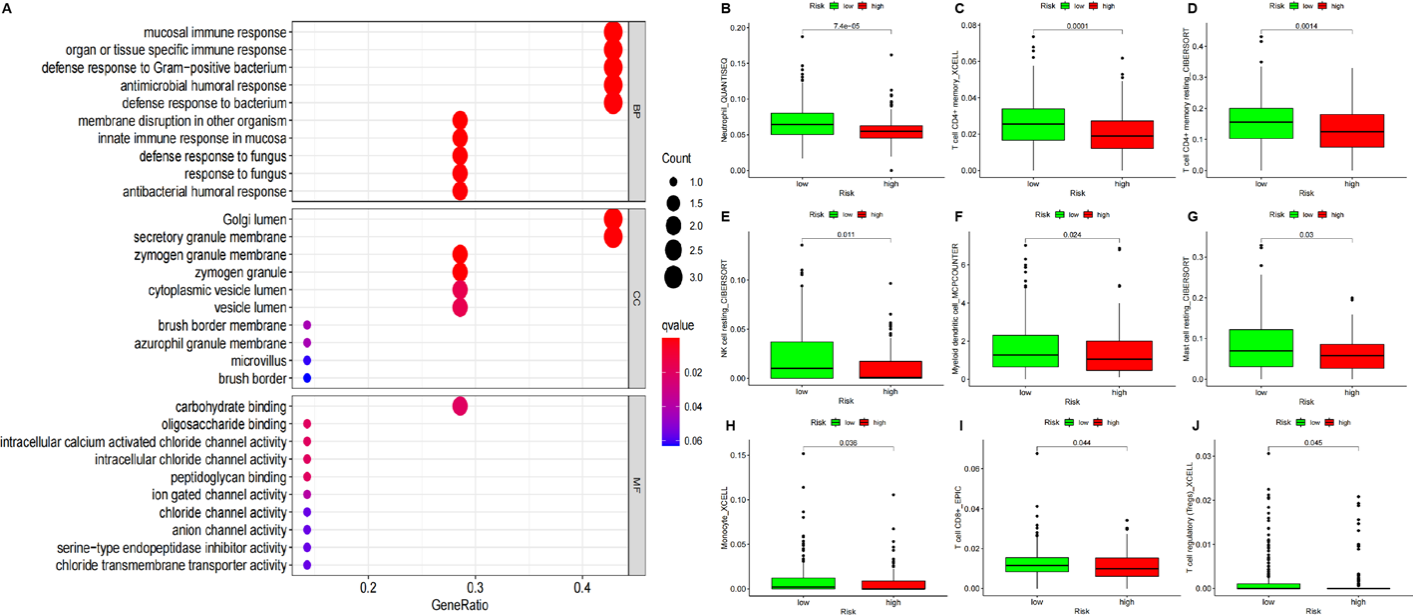
Functional differences based on grouping. **A** GO analysis, Color represents Pvalue, and the size of the balls shows gene number. MF,Molecular Function; CC, Cellular Component; BP, Biological Process. **B**–**J** Immune cell differential analysis, in order of preference are Neutrophil, T cell CD4 + memory, T cell CD4 + memory resting, NK cell resting, Myeloid dendritic cell, Mast cell resting, Monocyte, T cell CD8 + and T cell regulatory

To explore the functional differences in genes between high-low risk groups, we performed GO (Gene Ontology) functional enrichment analysis. Firstly, we used Wilcoxontest analysis to screen 7 differential genes (DEFA6, DEFA5, SPINK4, ITLN1, CLCA1, PIGR, ZG16), all of which were down-regulated in the high-risk group. We found that mucosal immune response、organ or tissue specific immune response、defense response to Gram-positive bacterium、antimicrobial humoral response and defense response to bacterium were most significant among the BP (Biological Process) terms; Golgi Lumen and secretory granule membrane were most significant among the CC(Cellular Component) terms; carbohydrate binding was most significant among the MF (Molecular Function) terms (Fig. 5A). Then, we analysed 51 immune cell samples from patients for differences between high and low risk groups. The data included immune cells from different algorithms (e.g. TIMER, CIBERSORT, CIBERSORT-ABS, QUANTISEQ, MCPCOUNTER, XCELL, and EPIC) to extract the content. Nine of these immune cells were found to differ between the high and low risk groups. In descending order of variability they were: Neutrophil (*p* = 0.000075), T cell CD4 + memory (*p* = 0.0001), T cell CD4 + memory resting (*p* = 0.0014), NK cell resting (*p* = 0.011), Myeloid dendritic cell (*p* = 0.024), Mast cell resting (*p* = 0.03), Monocyte (*p* = 0.036), T cell CD8 + (*p* = 0.044), T cell regulatory (Tregs, *p* = 0.045) (Fig. 5B–J).

### Analysis of drug sensitivity between the high-risk group and the low-risk group determined by the model

**Fig. 6:**
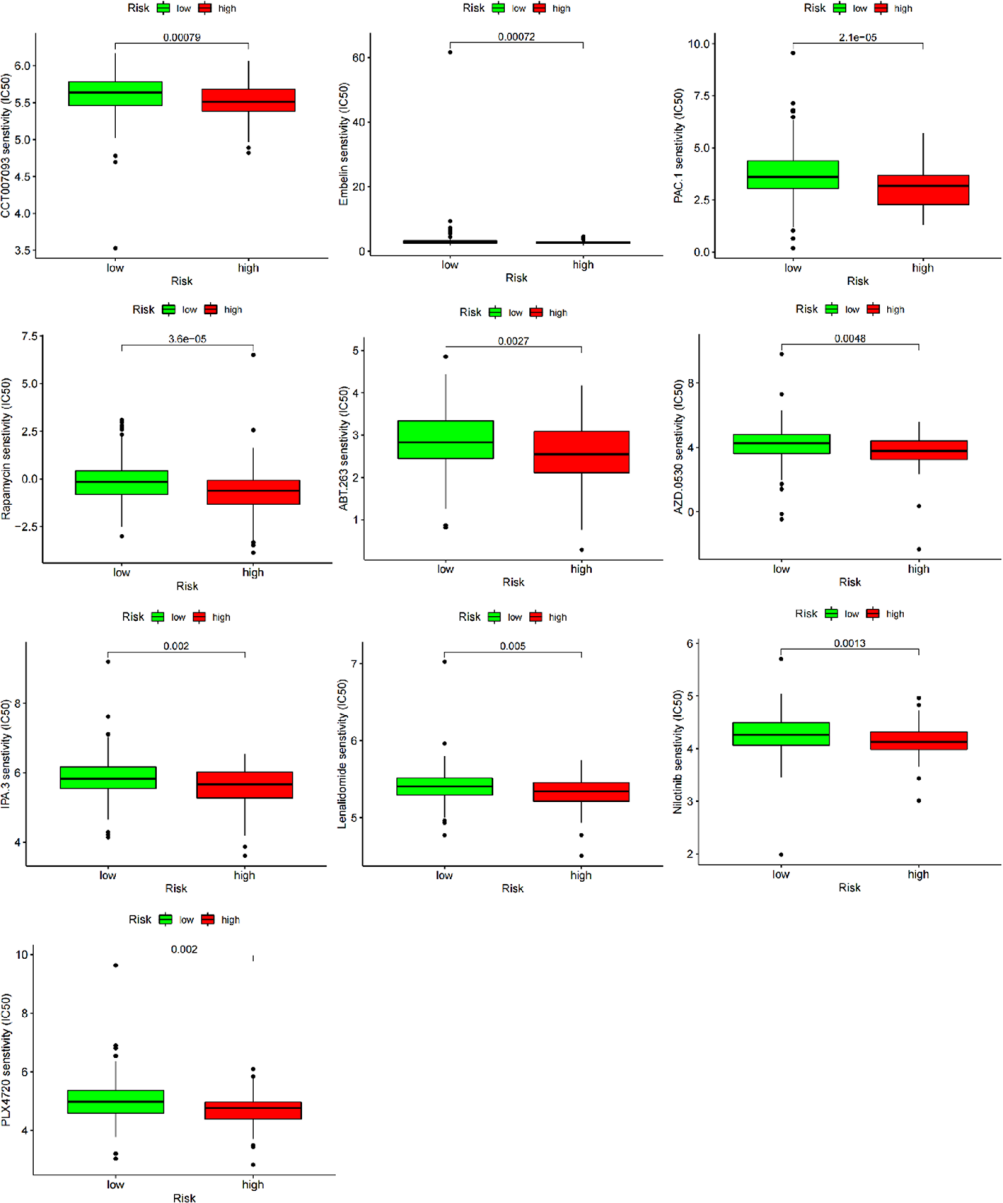
Analysis of drug sensitivity. CCT007093, Embelin, PAC1, and Rapamycin were the most sensitive (*p*-value < 0.001). ABT.263, AZD.0530, IPA.3, Lenalidomide, Nilotinib, PLX4720 were relatively sensitive (*p*-value < 0.01)

To explore drugs that contribute to colon cancer prognosis, we used the CGP database in combination with the high-risk group and the low-risk group for drug screening, we analyzed the sensitivity of 138 drugs. We found 10 sensitive drugs, among which CCT007093, Embelin, PAC1, and Rapamycin were the most sensitive (*p*-value < 0.001), ABT.263, AZD.0530, IPA.3, Lenalidomide, Nilotinib, PLX4720 were relatively sensitive (*p*-value < 0.01) (Fig. 6).

### CCK-8 assay effect of the most sensitive drugs on cell inhibition in COAD

**Fig. 7:**
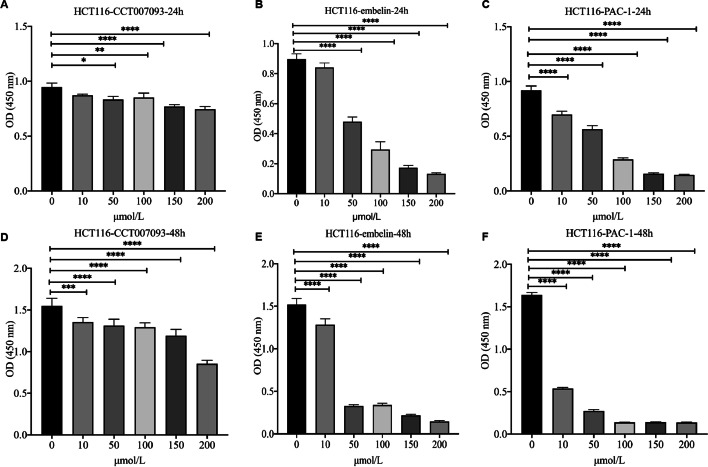
CCK-8 assay for the drug. **A** 24-h inhibitory capacity of CCT007093 drug. **B** 24-h inhibitory capacity of Embelin drug. **C** 24-h inhibitory capacity of the drug PAC-1. **D** 48-h inhibitory capacity of the CCT007093 drug. **E** 48-h inhibitory capacity of Embelin drug. **F** 48-h inhibitory capacity of PAC-1 drug. Where 0 mol/L is the control

Based on drug sensitivity analysis, we identified four highly sensitive drugs (*p* < 0.001), of which Rapamycin has already been shown to be a drug that can be used for COAD treatment, so we discuss the other three drugs experimentally. We used the CCK-8 assay to observe the inhibition of COAD cancer cells by three highly sensitive drugs (CCT007093, Embelin, PAC.1). The HCT116 cells was incubated in different drug solutions for 1 and 2 days. The inhibition of the drug was determined by measuring the OD (optical density) value, the lower the OD value the better the inhibition compared to the control 0 µmol/L. We plotted the cytostatic status of the three drugs on the first day of HCT116 (Fig. 7A–C), and the cytostatic status of the three drugs on the second day of HCT116 (Fig. 7D–F). We can visually see from these results that the three drugs have a significant inhibitory capacity and that the actual inhibitory capacity of the drugs is consistent with the strength of drug sensitivity (p-value magnitude) we have analysed (PAC-1 > Embelin > CCT007093).

### Nomogram model for personalized prediction based on riskscore and clinical characteristics

**Fig. 8:**
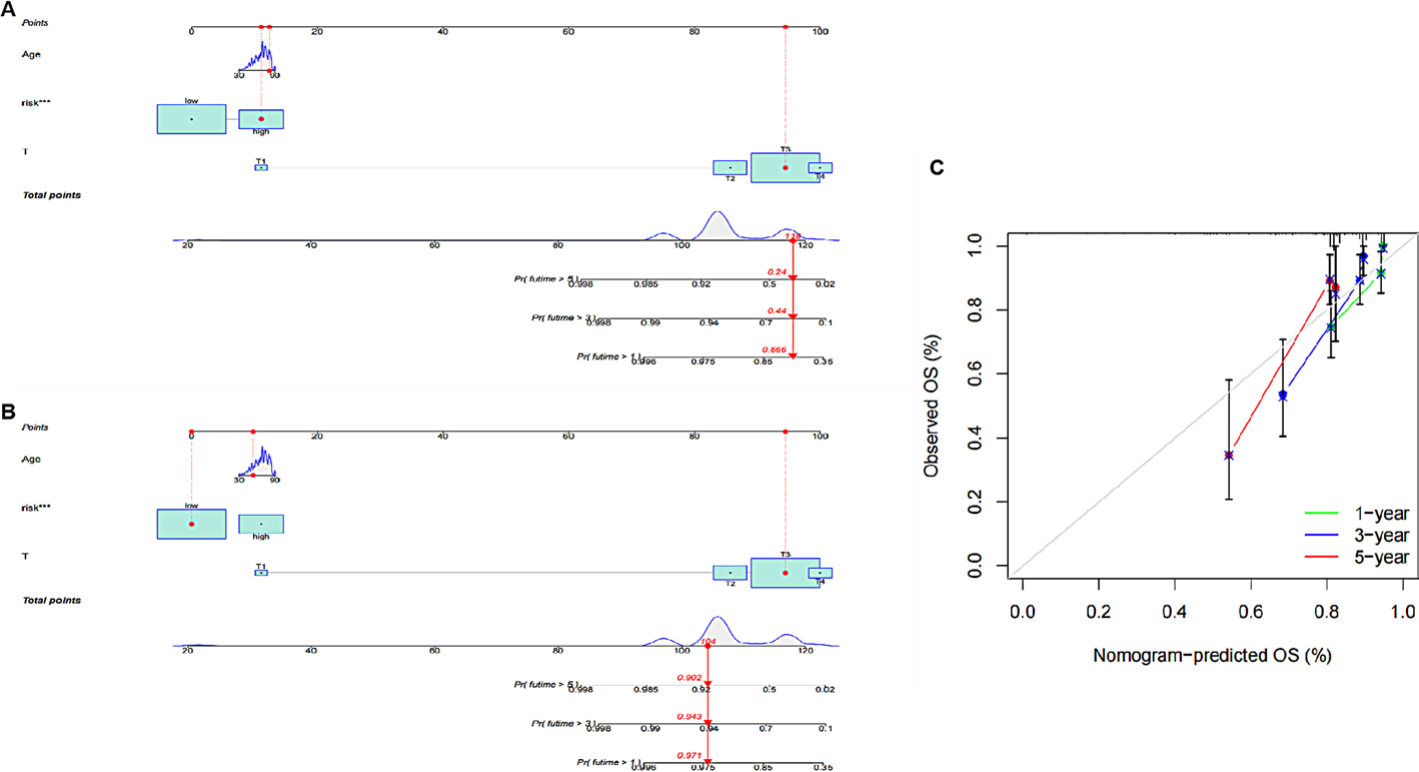
Nomogram individualized prediction model. C-index = 0.820 **A** Prediction of 1, 3 and 5 year prognostic survival probability of low-risk patients TCGA-A6-3807. **B** Prediction of 1, 3 and 5 year prognostic survival probability of high-risk patients TCGA-A6-2686. **C** Calibration curves of prognostic prediction models at 1, 3 and 5 years

In independent prognostic validation, age and T stage were also found to be predictive clinical features of colon cancer prognosis (*p*-value < 0.05), so we combined them with the model results to establish a Nomogram model to predict the survival probability of patients in 1, 3 and 5 years. We calculated the C-index of the model, and the value was 0.820. We predicted the prognostic survival of a low-risk patient TCGA-A6-3807 (Fig. 8A) and a high-risk patient TCGA-A6-2686 (Fig. 8B), among which the probability of prediction for low-risk patients was 0.971, 0.943 and 0.902, respectively. High risk patients were 0.666, 0.440, 0.240. The calibration degree of model 1, 3 and 5 years was analyzed through the calibration curve, and it was found that the prediction accuracy was high in the first year, and decreased as time went on (Fig. 8C).

## Discussion

COAD is a malignancy with a high fatality rate. Its etiology can be determined by diet [13], smoking, long-term use of NSAIDs (Non-steroidal Anti-inflammatory Drugs) and aspirin, other colorectal diseases, genetic predisposition, and metabolic syndromes [14]. Due to the heterogeneity of tumor molecules and the presence of many different genotypes, or subtypes, of cells, traditional methods cannot accurately distinguish related cancer risk conditions, such as tumor size and number of lymph nodes. Molecular markers have been studied extensively in COAD and can be a good predictor of risk. For instance, Haiti Chen et al. [15] developed and validated five immune gene models as auxiliary variables for predicting the prognosis of colon cancer.

Current research supports the hypothesis that lncRNAs have an important biological purpose. There is clear evidence that they are important in physiology, embryology, and development and have many new gene regulatory functions [16]. Moreover, their abnormal expression has been linked to a wide range of diseases, including cancers [17]. Compared with mRNA and microRNA, lncRNAs are located in both the cytoplasm and the nucleus, which further indicates their important role in epigenetic modification and gene regulation. Thus, the focus of molecular markers has shifted to lncRNAs [18]. Many previous studies have identified lncRNAs that are involved in the prognosis of COAD. For example, lncRNA xirP2-AS1 was found to be a favorable biomarker for colon cancer patients by analyzing 130 patients with COAD [19].

In our study, we obtained the gene expression profile of COAD from TCGA database and proposed the related lncRNA. After immune correlation analysis and differential analysis, the lncRNA was paired and then analyzed by univariate Cox, LASSO, and multivariate Cox analyses. We found eight pairs of lncRNAs that were closely related to patients with COAD and used the Cox risk regression model for verification. LASSO was utilized to prevent overfitting [20], and Cox was chosen as it offers the most accurate analysis of survival patterns [21]. We compared the clinical features (such as TMN stage, sex, and age) with the advantages of the model and found that the model had a high predictive accuracy. In addition, the Cox analysis showed that the model had independent prognostic ability. Gene pathways, immune cells and sensitive drugs were screened for differences between subgroups. Finally, taking into account individual patient differences, we used age, stage and risk score models to build nomogram models to predict the probability of patient survival at 1, 3 and 5 years.

We used pairwise pairing and orderly principle to establish lncRNA pairs, compared the expression level of the former lncRNA and the latter lncRNA, and assigned a value of 0 or 1. LncRNA pairs can reduce the influence of other data. It increases the universality of the model, only comparing the level of lncRNA in patients and avoiding the need for model batch correction for other clinical data, such as gene chip microarrays or PCR. We analyzed the 15 lncRNAs included in the model and found that nine lncRNAs were different in the high-risk and low-risk groups: AC008735.2, AC073283.1, AC104823.1, AL136115.2, AL442125.2, LINC02474, AC007128.1, AC021218.1, and PIK3IP1-AS1 (Additional file 2: Fig. S1).

Based on the high and low risk group results obtained by the model. We screened 51 different kinds of immune cells and found that Neutrophil, T cell CD4 + memory, T cell CD4 + memory resting, NK cell resting, Myeloid dendritic cell, Mast cell resting, Monocyte, T cell CD8 + and T cell regulatory have a high degree of Differences. In previous studies, 22 associated immune cells were established as models for the diagnosis and prognosis of stage I–III colon cancer [22]. This result improves the reliability of our model.

To further prove the reliability of the model. We used the model to analyze the *EGFR* gene and *APC* gene, finding that it differed between the high-risk and low-risk groups we classified (Additional file 2: Fig. S2). It is worth noting that *EGFR* has been proven to have good success as a drug-targeted therapy for colon cancer and has been used in clinical settings [23]. And in June 2021, it was proposed that the tumor suppressor gene *APC* is involved in the regulation of colon cancer. By inhibiting the mutation of *APC*, intestinal stem cells restore the competitiveness of wild-type cells, thereby improving the health of normal cells and limiting the proliferation and expansion of precancerous clones [24].

We analyzed 138 drugs, four of which were highly sensitive (*p*-value < 0.001), and six were sensitive (*p*-value < 0.01). Drug information is provided in Additional file 3. The mTOR inhibitor rapamycin found in previous studies can inhibit the prolongation of protein translation in *APC*-deficient tumor cells and can cause tumor cell growth stagnation [25]. Rapamycin has been widely used in the clinical treatment of colon cancer. In addition, we investigated the prediction of other drugs for the treatment of colon cancer, among the 10 drugs, Rapamycin [25], Embelin [26, 27] and ABT.263 [28, 29] are used to treat colon cancer, while CCT007093 [30, 31] is used to treat breast cancer. PAC.1 [32, 33] can be used to treat non-small cell lung cancer. AZD.0530 [34, 35] is used to treat gastric and ovarian cancer; IPA.3 [36, 37] can be used to treat metastatic prostate cancer and hepatocellular carcinoma; Lenalidomide [38, 39] can be used to treat lymphoma; Nilotinib [40] can be used to treat chronic myeloid leukemia; PLX4720 [41] can be used in the treatment of thyroid cancer. These drugs have not been studied in the treatment of colon cancer, so we proposed the possibility of using these drugs in colon cancer. In particular, we analysed the inhibitory capacity of the highly sensitive drugs CCT007093, Embelin and PAC. This was validated using HCT116 COAD cell lines and experiments using the CCK-8 method showed that these drugs exhibited extremely high inhibition with increasing concentrations and that the drugs inhibited in a manner consistent with the ranking of the sensitive drugs we analysed. This also demonstrates the accuracy of the prediction model.

Finally, we developed a nomogram model to predict the probability of survival at 1, 3 and 5 years for different patients with a C-index of 0.820. A number of nomogram decision models have been developed in previous studies, and Yuting Qiu et al. [42] developed a prognostic model by analysing Ferroptosis-Related LncRNAs, with a C-index of 0.801. The performance of our model was slightly improved compared to the prognostic model built directly using lncRNAs, and we processed the data to take into account the differences in results obtained from different measurements and to avoid the effect of batch correction, we paired the lncRNAs and only analysed the relative content levels.

In conclusion, our study proposed a prediction model of colon cancer prognosis based on eight pairs of lncRNAs and found drugs sensitive to COAD based on this model. However, the connection between lncRNAs and drugs is not clear at present, and this aspect will be investigated in future research studies.

## Methods

### Patients and datasets

**Fig. 9:**
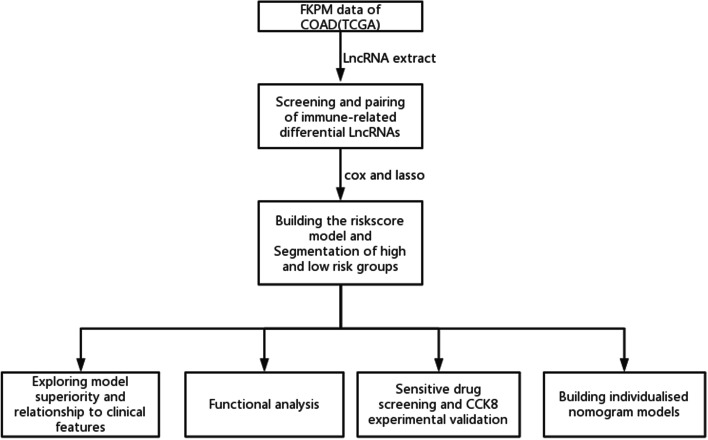
Construction of a prognostic model for COAD based on ncRNA pairs and the drug screening process. Based on gene expression data in FPKM format for COAD in TCGA, we screened eight prognosis-related ncRNA pairs, established a prognostic model risk score using cox and lasso, assessed the superiority of the model and analysed the functional differences between model subgroups. Among 138 drugs screened for potential use in the treatment of high-risk COAD and subjected to CCK-8 cellular assays, an individualised nomogram model was finally developed for clinical decision making

We downloaded the FPKM data of 437 COAD samples from 385 patients, including 398 tumors samples and 39 normal samples with their corresponding clinicopathological information from TCGA database (https://portal.gdc.cancer.gov/projects/TCGA-COAD, Data Release 27.0–29 October 2020). The human gene annotation file (gencode.v29.Annotation.gtf.gz) was downloaded from Gencode (https://www.gencodegenes.org/human/release_29.html). The immune genetic file (GeneList.xls) was downloaded from the IMMPORT system (https://www.immport.org/shared/genelists). The immune infiltration file (infiltration_estimation_for_tcga.csv) was downloaded from the TIMER2.0 [43] (http://timer.comp-genomics.org/infiltration_estimation_for_tcga.csv.gz). All methods were carried out in accordance with relevant guidelines and regulations, and Our code is open source on github (https://github.com/lpc-97/COAD.git). The overall flow chart is shown in Fig. 9.

### Identification of immune gene-associated lncRNAs and establishment of lncRNA pairs

An Ensembl ID tool was used to convert the original sequencing data into gene names, while mRNA and lncRNA in the original sequencing data were extracted for downstream analysis. The expression levels of 1711 immune genes corresponding to 385 patients with COAD were extracted. A total of 1229 lncRNAs associated with immune genes were analyzed and compared using the Pearson’s correlation coefficient (PCC (Pearson Correlation Coefficient) > 0.4, *p*-value < 0.001). Finally, 226 lncRNAs (FDR (False Discovery Rate) < 0.05, |${\mathrm{log}}_{2}FC (log2fold change)$|> 1) were differentiated and identified by using the limma package of R [44], and We used pairwise pairing and orderly principle to establish lncRNA pairs, compared the expression level of the former lncRNA and the latter lncRNA, and assigned a value of 0 or 1. In this way, we constructed 16,232 lncRNA pairs.

### Establishment of a prognostic model of immune-related lncRNAs

To improve the credibility of the prognostic model, 385 patients with colon cancer were randomized into trial sets (n = 265) and validation sets (n = 120). Where after removing samples without survival features, the training set totals 249 and the validation set totals 114. The clinical characteristics of the subgroups are shown in the Table 2. We can see no significant differences between the subgroups. The expression level of immune gene-related lncRNAs and prognosis follow-up data of patients were integrated. A univariate Cox analysis (*p*-value < 0.001) was performed for each lncRNA pair to screen out the lncRNA pairs associated with prognosis, and we then used LASSO to further screen prognostic lncRNAs. A multivariate Cox regression analysis was performed on the screened lncRNAs to further screen out the lncRNA pairs used to construct the prognostic risk assessment formula for COAD patients, and the corresponding lncRNA pairs' coefficients were calculated. So we ended up with eight lncRNA pairs. Using the prognostic risk score of the patients, the optimal cut-off (Youden index = sensitivity − (1 − specificity)) was calculated, and the patients were divided into high-risk and low-risk groups.

**Table 2:** Clinical characteristics of the training and validation sets

Clinical	Type	Total	Validation	Train	*p* value
Age	< = 65	153 (42.15%)	52 (45.61%)	101 (40.56%)	0.4294
	> 65	210 (57.85%)	62 (54.39%)	148 (59.44%)	
Gender	Female	167 (46.01%)	48 (42.11%)	119 (47.79%)	0.3706
	Male	196 (53.99%)	66 (57.89%)	130 (52.21%)	
Stage	Stage I	63 (17.36%)	21 (18.42%)	42 (16.87%)	0.9773
	Stage II	140 (38.57%)	44 (38.6%)	96 (38.55%)	
	Stage III	98 (27%)	31 (27.19%)	67 (26.91%)	
	Stage IV	51 (14.05%)	15 (13.16%)	36 (14.46%)	
	Unknow	11 (3.03%)	3 (2.63%)	8 (3.21%)	
T	T1	9 (2.48%)	2 (1.75%)	7 (2.81%)	0.3358
	T2	65 (17.91%)	20 (17.54%)	45 (18.07%)	
	T3	249 (68.6%)	74 (64.91%)	175 (70.28%)	
	T4	39 (10.74%)	17 (14.91%)	22 (8.84%)	
	Unknow	1 (0.28%)	1 (0.88%)	0 (0%)	
M	M0	272 (74.93%)	87 (76.32%)	185 (74.3%)	0.8425
	M1	51 (14.05%)	15 (13.16%)	36 (14.46%)	
	Unknow	40 (11.02%)	12 (10.53%)	28 (11.24%)	
N	N0	217 (59.78%)	67 (58.77%)	150 (60.24%)	0.8998
	N1	84 (23.14%)	26 (22.81%)	58 (23.29%)	
	N2	62 (17.08%)	21 (18.42%)	41 (16.47%)	

### Analysis of functional differences based on model

First, we analysed differences in gene pathways between model subgroups using GO methods. We then analysed the differences in prognosis-related immune cells, From the immune infiltration file, a total of 51 immune cells were analyzed (such as B cell, T cells, NK cell, Mast cell, Macrophage, Monocyte and their subtypes and some immune-related cells), 9 immune cells with differences were identified (*p*-value < 0.05).

### Analysis of sensitive drug based on model

A sensitivity analysis was performed on 138 drugs. First, the CGP database and the predicted expression matrix were combined to remove the genes with low expression levels. All remaining genes were used as predictors with the drug sensitivity (IC50) values of the drug in question as the outcome variable. When the IC50 value of the high-risk group is lower than that of the low-risk group and there is a significant difference between the high- and low-risk groups, this indicates that the drug is effective for the patient. Thus, this information was finally combined with the model risk results to further identify the sensitive drugs with a low and differentiated IC50 at high risk. The usage method is integrated in the ‘pRRophetic’ R package [45].

### Analysis of the inhibitory capacity of highly sensitive drugs

We used HCT116 cell lines for CCK-8 analysis, each, washing the cells 3 times with PBS, adding 2 mL of trypsin, digesting for 1–2 min, discarding the trypsin, gently blowing the cells with a disposable pipette to dislodge them and adding to a centrifuge tube containing 2 mL of culture medium to make a suspension. Aspirate 10 µL of the single cell suspension and place on a hemocytometer plate for cell counting, adjusting the concentration of the single cell suspension to 5 × 104 cells/ mL. After 24 h, the 96-well plates were removed and 100 µL of fresh drug solution at different concentrations (0, 10, 50, 100, 150, 200 µmol/L) was added to each 96-well plate. 3 replicate wells were set up for each group and incubated in the cell culture incubator. OD values were measured on days 1 and 2 after addition. The ability of the drug to inhibit COAD cancer cells was determined by analysing the absorbance of the cells.

### Establishment of a nomogram model for individualized prediction based on high and low risk groups

Finally, in order to better participate in clinical decision-making, a Nomogram model is established to predict the survival probability of patients in 1, 3 and 5 years, and the accuracy of the nomogram model is demonstrated by the calibration curve using the consistency index evaluation model.

### Visualization analysis based on prognostic model

The survival curves of patients with COAD were plotted by using the survival and survminer R package, and the P-values were calculated to perform LASSO and visualization using the glmnet program package. Models, post-independent analyses, and visualization of clinical traits were developed using survival and forest maps. We used the survivalROC function to draw projections for 1, 3, and 5 years, for some clinical traits of survival, for the ROC, and to calculate the AUC. We used the ComplexHeatmap package to draw clinically relevant heat maps and the ggpubr package to draw a boxplot of immune cell, gene, and drug correlation analyses. The Prophetic package was used for drug sensitivity analysis.

### Statistical method

Data analysis software R (version 4.0.4) and RStudio (version 1.4.1103) were used to conduct data analyses and generate statistical charts. The Pearson’s correlation coefficient was used to calculate the correlation, and PCCs > 0.4 and *p*-value < 0.001 were considered to be correlated. A univariate Cox analysis was used to identify the lncRNA pairs, associated with prognosis, and the test level α = 0.001. A multivariate Cox was used to model, and the test level α = 0.1. Univariate and multivariate Cox analyses were used to determine whether or not the prognostic risk score and other clinical information were associated with prognosis, and the test level α = 0.001. We set the enrichment differential gene screen fdr < 0.05 and |log2FC)|> 1. The data from the CCK-8 experiment were statistically analysed using Graphpad prism 8.4 software. The statistical data were expressed as (One-way ANOVA) between groups, and the experiment was repeated three times, with differences considered statistically significant at *p < 0.05.

## Supplementary Information

**Additional file 1**. 1229 lncRNAs screens associated with immune genes.

**Additional file 2**. Figure: Differences in genes in the high- and low-risk groups.

**Additional file 3**. Detailed information on 10 sensitive drugs.

## Data Availability

The datasets analysed during the current study are available in the TCGA (https://portal.gdc.cancer.gov), GENCODE (https://www.gencodegenes.org), IMMPORT (https://www.gencodegenes.org), TIMER2.0 (http://timer.comp-genomics.org).

## Abbreviations

lncRNA: Long non-coding RNAs
TCGA: The Cancer Genome Atlas
COAD: Colon adenocarcinoma
COX: Proportional hazards model
CGP: Cancer genome project
PCCs: Pearson correlation coefficient
FDR: False discovery rate
log_2_FC: Log_2_fold change
IC50: Half maximal inhibitory concentration
OD: Optical density
