# Supplementary material for: Prognostic risk assessment model and drug sensitivity analysis of colon adenocarcinoma (COAD) based on immune-related lncRNA pairs
Source: BMC Bioinformatics. 2022 Oct 18;23:435. doi: 10.1186/s12859-022-04969-4 (PMC9579580; doi:10.1186/s12859-022-04969-4)
Supplement: Supplementary file 2 — Additional file 2. Figure: Differences in genes in the high- and low-risk groups. [file 12859_2022_4969_MOESM2_ESM.docx]

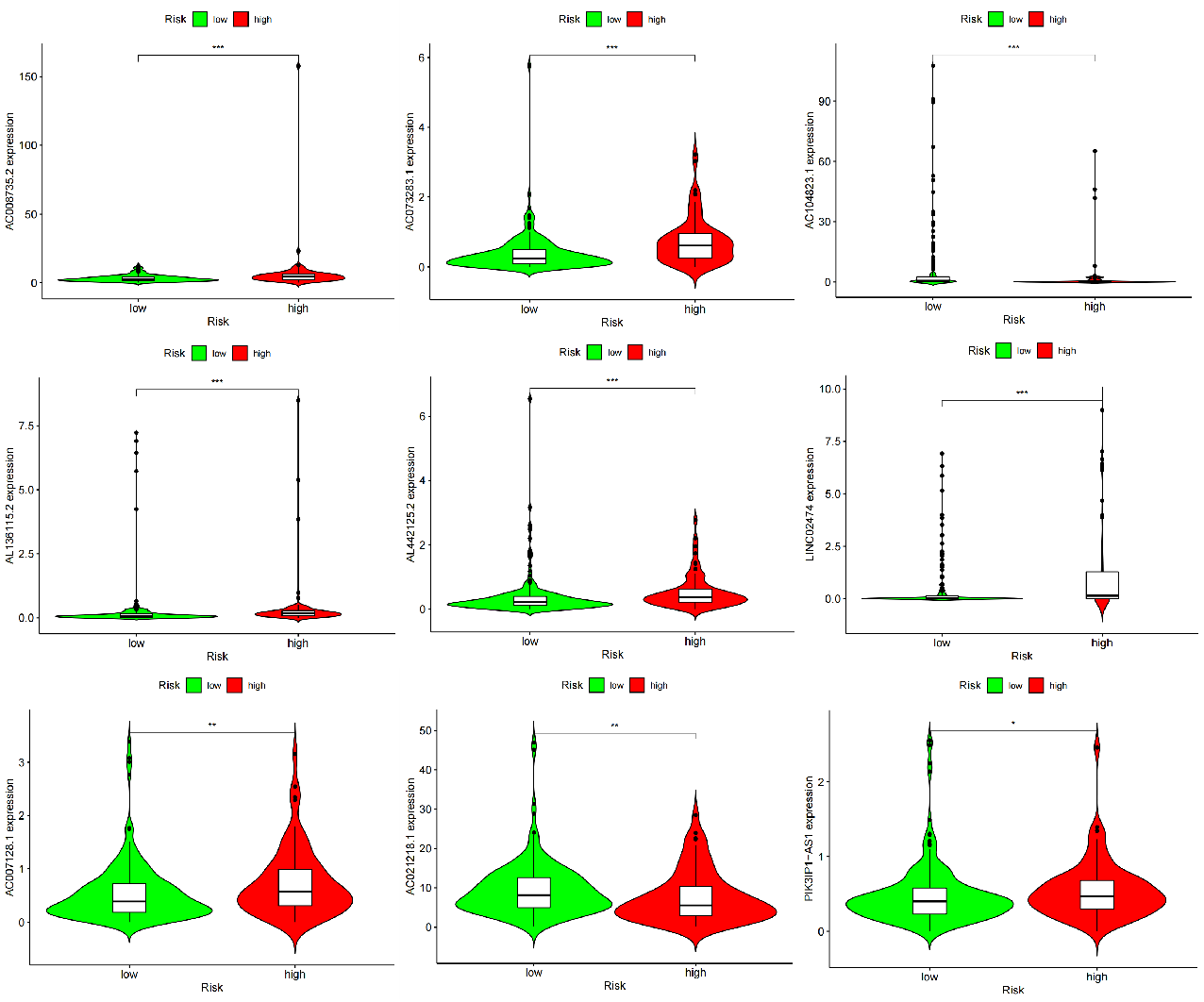


Supplementary Figure 1. Analysis of the single lncRNA in the model. In the model, nine lncRNAs showed differences among the high- and low-risk groups. In order, they were AC008735.2, AC073283.1, AC104823.1, AL136115.2, AL442125.2, LINC02474, AC007128.1, AC021218.1, and PIK3IP1-AS1. (* : p < 0.05, ** : p < 0.01, *** : p < 0.001).


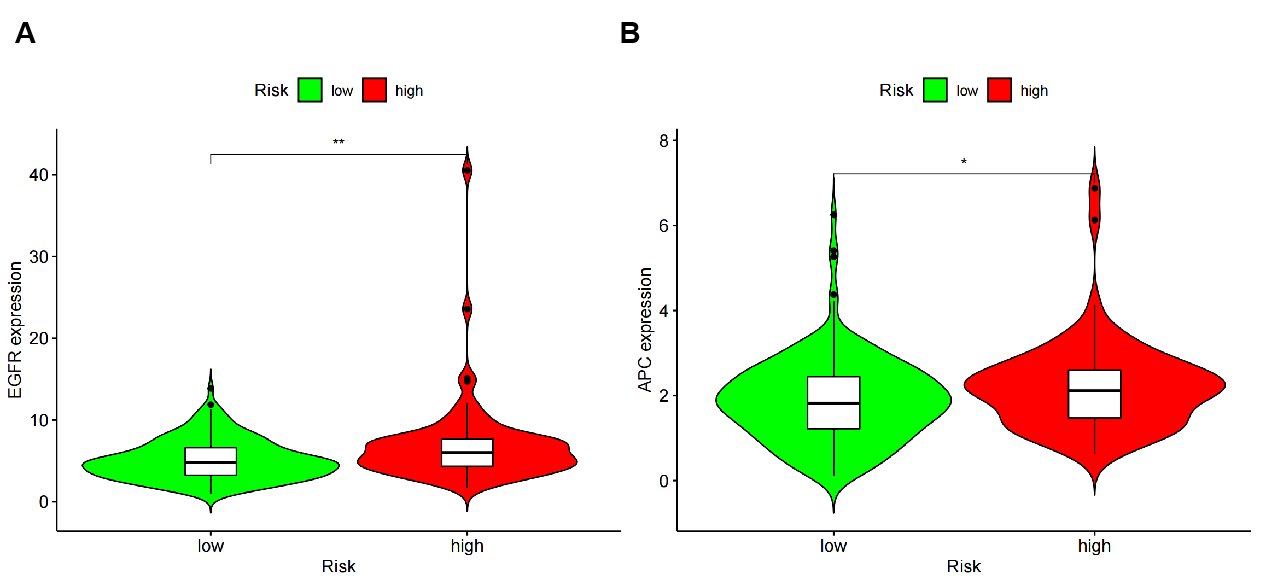


Supplementary Figure 2. Analysis of differences in EGFR and APC between high- and low-risk groups. Left, EGFR;Right, APC.(* : p < 0.05, ** : p < 0.01).
