# Supplementary material for: Prognostic risk assessment model and drug sensitivity analysis of colon adenocarcinoma (COAD) based on immune-related lncRNA pairs
Source: BMC Bioinformatics. 2022 Oct 18;23:435. doi: 10.1186/s12859-022-04969-4 (PMC9579580; doi:10.1186/s12859-022-04969-4)
Supplement: Supplementary file 3 — Additional file 3. Detailed information on 10 sensitive drugs. [file 12859_2022_4969_MOESM3_ESM.docx]

Drug

## Structure diagram of the drug from PUBCHEM database (https://pubchem.ncbi.nlm.nih.gov/)

1. Rapamycin (ID: 5284616)


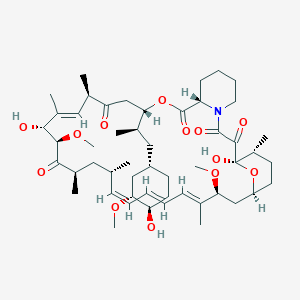


Rapamycin is a specific mTOR inhibitor. mTORC1 has been observed to increase in activation in many human cancers, and rapamycin forms a gain-of-function complex with a binding protein (FKBP12) specifically as an mTORC1 inhibitor and is used in a variety of cancers. William J Faller^[25]^ et al. proposed that mTORC1-mediated eEF2 kinase inhibition is required for the proliferation of APC-deficient cells. Thus the mTOR inhibitor rapamycin inhibits the prolongation of protein translation in apc-deficient tumor cells and can lead to tumor cell growth arrest. And it is noted that inhibition of translation elongation using existing clinically approved drugs such as rapalogs would provide significant therapeutic benefit for patients at high risk of developing colorectal cancer. In our study, Rapamycin was significantly different between the high- and low-risk groups (p=0.000036), which is also consistent with the results of the between studies.

## Embelin (ID: 3218)


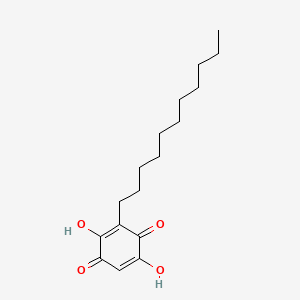


Embelin is a quinone isolated from dwarf ground tea and is an inhibitor of the X chromosome inhibitor of apoptosis (XIAP). embelin has a wide range of applications in cancer prevention and treatment, and many studies have identified a variety of biological properties of embelin, including antitumor, anti-inflammatory, analgesic, anti-diabetic, and antioxidant effects, which have been evaluated for use in the treatment of a range of cancer types, suggesting that the use of embelin-induced autophagy may be an important strategy for cancer prevention. you-Jin Lee[26] et al. showed that embelin exhibited anticancer activity against Ca9-22 through autophagy and apoptosis. In turn, it may contribute to oral cancer treatment and provide useful information for the development of new therapeutic agents. Yun Dai^[27]^ et al. found that Embelin reduces colitis-associated tumorigenesis by limiting IL-6 / STAT3 signaling, suggesting that Embelin may be a potential agent for the prevention and treatment of CAC. In our study, Embelin showed a significant difference between high and low risk groups (p=0.00072), which is also consistent with the results of previous studies.

## 3. ABT-263 (ID: 24978538)


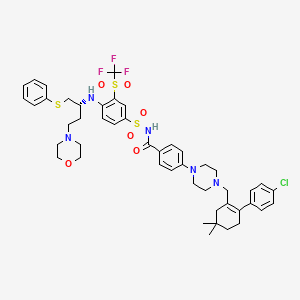


Navitoclax (ABT-263) is a potent inhibitor of Bcl-xL, Bcl-2 and Bcl-w. Christin Tse^[28]^ et al. proposed that ABT-263 is a potent, orally bioavailable poor BH3 mimetic and that oral administration of ABT-263 alone induced complete tumor regression in small cell lung cancer and acute lymphoblastic leukemia xenograft models. Huanjie Shao^[29]^ et al. evaluated the interaction between apigenin and ABT-263 in colon cancer cells. The synergistic effect of apigenin and ABT-263 on apoptosis of colon cancer cells was found. Finally, a novel strategy for the enhancement of ABT-263-induced antitumor activity in human colon cancer cells by apigenin through the inhibition of Mcl-1, AKT and ERK pre-modulators is proposed. In our study, we found a difference in ABT-263 between high and low risk groups (p=0.0027), which also provides the possibility of ABT-263 as a therapeutic agent for colon cancer.

**4. CCT007093 (ID: 2314623)**

**
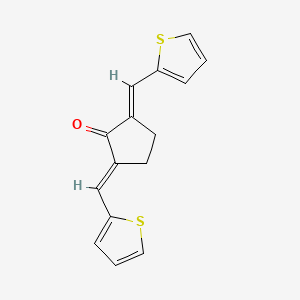
**

CCT007093 is a potent PPM1D (WIP1) inhibitor. Joshua A Bauer^[30]^ et al. used CCT007093 in combination with paclitaxel and found that the conjugate could produce synergistic inhibition in four paclitaxel-resistant triple-negative breast cancer (TNBC) cell lines. Lee JS^[31]^ et al. found that CCT007093 could selectively promote apoptosis in breast cancer cells and apoptosis in skin-transformed ectopic Wip1-expressing keratinocytes. CCT007093 is currently often studied for breast cancer and less frequently for colon cancer, and in our study it was found to be significantly different between high- and low-risk tissues (p=0.00079), so we propose that CCT007093 is possible for the treatment of patients with high-risk colon cancer.

## 5. PAC-1 (ID: 135421197)


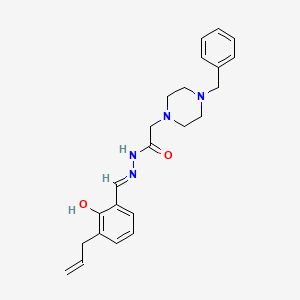


PAC-1 is a potent activator of procaspase-3 (zymogen-3), the first known small molecule that directly activates zymogen-3 as caspase-3. Dan Lu^[32]^ et al. proposed PAC-1 as an epigenetic immunomodulator and highlighted that targeting PAC1 is important in cancer immunotherapy. yuan-Li Zhou^[33]^ et al. used mirna, proposed that MiR-34c-3p inhibits proliferation and invasion of non-small cell lung cancer (NSCLC) through inhibition of the PAC1/MAPK pathway. Previous studies have shown that PAC-1 can be used in the treatment of tumors, and in our study, PAC-1 was significantly different between high- and low-risk groups (p=0.000021), so PAC-1 may also be used to treat patients with high-risk colon cancer.

## AZD-0530 (ID: 10302451)


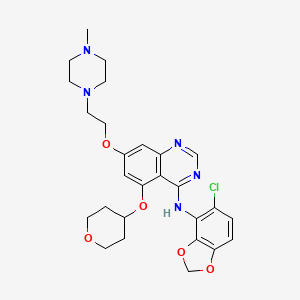


Saracatinib (AZD0530) is a potent Src inhibitor, and Hyun-Jin Nam^[34]^ et al. noted that Src is non-receptor tyrosine kinase involved in crosstalk and mediation of many signaling pathways that promote cell proliferation, adhesion, invasion, migration, and tumorigenesis. Src activity is increased in patients with gastric cancer, and therefore, this factor has been identified as a promising therapeutic target for cancer therapy. By investigating the antitumor effects of saracatinib alone or in combination with other drugs, new strategies for the treatment of gastric cancer are proposed. Amanda L Jackson^[35]^ et al. found that saracatinib has a good inhibitory effect on angiogenesis in ovarian cancer. In our study, we found a difference in AZD0530 between high- and low-risk groups (p=0.0048), which also opens up the possibility of AZD0530 as a therapeutic agent for colon cancer.

## IPA-3 (ID: 521106 )


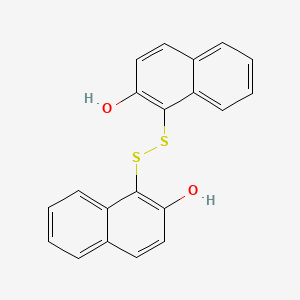


IPA-3 is a selective, non-ATP-competitive Pak1 inhibitor. Arti Verma^[36]^ et al. clearly proposed IPA-3 as a potential drug for the treatment of bone metastatic PCa by studying the efficacy of IPA-3 on metastatic prostate cancer (PCa) and found that IPA-3 significantly inhibited the proliferation and motility of mouse metastatic PCa cells in vitro. leo Lap- Yan Wong^[37]^ et al. proposed that IPA-3 not only inhibited the growth of hepatocellular carcinoma (HCC) cells, but also inhibited the metastatic potential of HCC cells. suggesting that IPA-3 has potential clinical efficacy. In our study, we found that IPA-3 was differential between high- and low-risk groups (p=0.002), which also provides the possibility of IPA-3 as a therapeutic agent for colon cancer.

## Lenalidomide (ID: 216326)


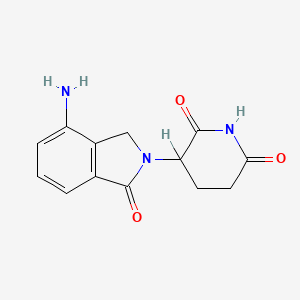


Lenalidomide is a TNF-α secretion inhibitor, and Graham H Jackson^[38]^ et al. found that Lenalidomide maintenance therapy significantly improved progression-free survival in patients with newly diagnosed multiple myeloma, with reliable clinical efficacy.John P Leonard^[39]^ et al. found through a drug treatment study in inert lymphoma Lenalidomide nicely improved the efficacy of rituximab in patients with recurrent inert lymphoma. In our study, we found a difference in Lenalidomide between high- and low-risk groups (p=0.005), which also opens up the possibility of Lenalidomide as a therapeutic agent for colon cancer.

## Nilotinib (ID: 644241)


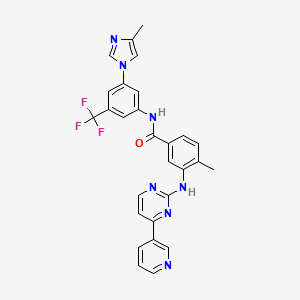


Nilotinib is a specific BCR-ABL inhibitor and many previous studies have found a large role for Nilotinib in chronic myeloid leukemia.Hagop M Kantarjian^[40]^ et al. followed patients with newly diagnosed chronic myeloid leukemia (CML) in the chronic phase for ≥10 years and found that patients treated with Nilotinib had the highest overall efficacy and safety. In our study, we found a difference in Nilotinib between high- and low-risk groups (p=0.0013), which also opens up the possibility of Nilotinib as a therapeutic agent for colon cancer.

## 10. PLX4720 (ID: 24180719)


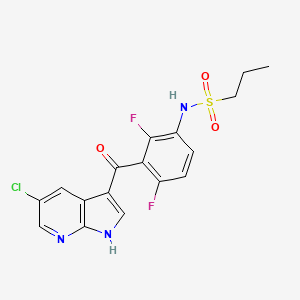


PLX-4720 is a potent, selective inhibitor of B-RafV600E. Francesca Coperchini^[41]^ et al. investigated the effect of PLX4720 on basal and TNF- α -induced CXCL8 secretion in thyroid cells and showed that PLX4720 inhibits CXCL8 secretion in BRAFV600E mutant thyroid cancer cells. PLX-4720 has been shown to be useful in the treatment of thyroid cancer. In our study, we found a difference in PLX-4720 between high and low risk groups (p=0.002), which also provides the possibility of PLX-4720 as a therapeutic agent for colon cancer.
